# Population-Based Serosurvey for Severe Acute Respiratory Syndrome Coronavirus 2 Transmission, Chennai, India 

**DOI:** 10.3201/eid2702.203938

**Published:** 2021-02

**Authors:** Sriram Selvaraju, Muthusamy Santhosh Kumar, Jeromie Wesley Vivian Thangaraj, Tarun Bhatnagar, Velusamy Saravanakumar, Chethrapilly Purushothaman Girish Kumar, Krithikaa Sekar, Ezhilarasan Ilayaperumal, Ramasamy Sabarinathan, Murugesan Jagadeesan, Masanam Sriramulu Hemalatha, Manoj Vasant Murhekar, the Chennai COVID Sero-Surveillance Team

**Affiliations:** Indian Council of Medical Research–National Institute for Research in Tuberculosis, Chennai, India (S. Selvaraju, K. Sekar, E. Ilayaperumal);; ICMR–National Institute of Epidemiology, Chennai (M. Santhosh Kumar, J.W. Vivian Thangaraj, T. Bhatnagar, V. Saravanakumar, C.P. Girish Kumar, R. Sabarinathan, M.V. Murhekar);; Greater Chennai Corporation, Chennai (M. Jagadeesan, M.S. Hemalatha)

**Keywords:** respiratory infections, severe acute respiratory syndrome coronavirus 2, SARS-CoV-2, SARS, COVID-19, coronavirus disease, zoonoses, viruses, coronavirus, IgG, transmission, serosurvey, Chennai, India

## Abstract

We conducted a cross-sectional survey to estimate the seroprevalence of IgG against severe acute respiratory syndrome coronavirus 2 in Chennai, India. Among 12,405 serum samples tested, weighted seroprevalence was 18.4% (95% CI 14.8%–22.6%). These findings indicate most of the population of Chennai is still susceptible to this virus.

On August 15, 2020, India had the third highest number of coronavirus disease (COVID-19) cases globally (*1*). The Indian state of Tamil Nadu reported 332,105 cases and 5,641 deaths on August 15, and ≈35% cases were from the state capital, Chennai (*2*). Administratively, Greater Chennai Corporation (GCC) is divided into 15 zones that are further divided into 200 wards with populations ranging from 4,400–104,558 (*3*). The total population of GCC is 7.1 million and 31% of the population resides in slums.

As a part of nationwide containment strategy, Chennai was under lockdown beginning March 25, 2020; beginning May 4, the lockdown was relaxed in a phased manner. Wearing facemasks in public has been mandatory since April 13. However, the number of COVID-19 cases has been increasing in Chennai since May. 

Serologic surveys can provide a comprehensive picture of community spread of severe acute respiratory syndrome coronavirus 2 (SARS-CoV-2), the causative agent of COVID-19 (*4*). During the first week of May, the unweighted seroprevalence in Chennai was 2% (*5*). We conducted a community-based serosurvey in July 2020, to estimate the seroprevalence of SARS-CoV-2 in GCC.

## The Study

We conducted a household-based cross-sectional survey among usual residents >10 years of age in GCC. To estimate a seroprevalence of 2%, with 20% relative precision, design effect of 2.5, and 95% CI, we needed a sample size of 11,710 persons, which we rounded to 12,000. We used a multistage cluster sampling method to select the survey participants. In the first stage, we selected 51 wards by using probability proportion to population size method. In the second stage, we randomly selected 6 streets from each ward from which to recruit participants. The survey team selected a random starting point in each street and visited contiguous households to enroll >40 consenting persons >10 years of age. When no one was home or household members were unavailable, the team proceeded to the next house and completed the survey until >40 persons were enrolled from each street. We included all eligible persons in the household who consented.

After obtaining written consent from persons >18 years of age, and assent and parental or guardian approval from persons <18 years of age, we interviewed participants to collect information. We used the Open Data Kit application (https://opendatakit.org) to collect sociodemographic details, and information on exposure to laboratory-confirmed COVID-19 case, history of COVID-19 symptoms in the past 3 months, and COVID-19 testing status. 

After the interview, we collected 3–5 mL of venous blood from each participant into BD Vacutainer Blood Collection Tubes (Becton Dickenson, https://www.bd.com). We later tested serum samples for IgG against SARS-CoV-2 by using SARS-CoV-2 IgG immunoassay (Abbott, https://www.corelaboratory.abbott) (Appendix) (*6*). The study protocol was approved by the Institutional Ethics Committee of ICMR-National Institute of Epidemiology.

We analyzed the data to estimate weighted seroprevalence of SARS-CoV-2 and 95% CI by using appropriate sampling weights. We further adjusted the seroprevalence for assay characteristics (*6*). We estimated the total number of SARS-CoV-2 infections among persons >10 years of age and infection-to-case ratio (ICR) (Appendix).

The survey teams visited 7,234 households from 321 streets across 15 zones. Of the 18,040 residents >10 years of age in the visited households, 14,839 (82.3%) were available at the time of survey, among whom 12,405 (83.6%) consented to participate (Appendix Table 1). The mean age of survey participants was 41.1 years (SD 17.3 years); 52.7% were female and 47.3% were male. Among 496 (4%) persons who reported prior reverse transcription-PCR (RT-PCR) testing for COVID-19, 119 (24%) reported testing positive (Table 1).

**Table 1:** Characteristics of 12,405 participants in a SARS-CoV-2 serosurvey, Chennai, India, July 2020*

Characteristics	No. (%)
Age, y, n = 12,319	
10–19	1,473 (12.0)
20–29	2,105 (17.1)
30–39	2,353 (19.1)
40–49	2,353 (19.1)
50–59	1,927 (15.6)
>60	2,108 (17.1)
Sex, n = 12,319	
M	5,785 (47.0)
F	6,493 (52.7)
Transgender	41 (0.3)
History of respiratory symptoms, n = 12,248	175 (1.4)
Symptomatic persons seeking medical care, n = 175	121 (69.1)
Hospitalization among persons seeking medical care, n = 121	71 (58.7)
Reported contact with COVID-19 case, n = 12,248	173 (1.4)

*Among 12,405 persons enrolled in the survey, age and sex data were not available for 86 participants. COVID-19, coronavirus disease; SARS-CoV-2, severe acute respiratory syndrome coronavirus 2.

Among 12,405 serum samples tested, 2,673 were positive for IgG , a weighted prevalence of 18.7% (95% CI 15.1%–22.9%). After adjusting for the test sensitivity and specificity, seroprevalence was 18.4% (95% CI 14.8%–22.6%) (Table 2). The weighted seroprevalence was higher among female participants (20.6%, 95% CI 16.7%–25.3%) than male participants (16.6%, 95% CI 13.2%–20.6%) (p<0.001). Weighted seroprevalence was lowest among persons >60 years of age (13.4%, 95% CI 10.3%–17.4%) than younger persons (p = 0.001) (Table 2). We retested 100 seronegative and 40 seropositive samples and results were concordant.

**Table 2:** Characteristics of persons with IgG against SARS-CoV-2, Chennai, India, July 2020*

Characteristics	No. tested	No. positive	Unadjusted seroprevalence, % (95% CI)	Weighted seroprevalence, % (95% CI)	p value	Test performance-adjusted seroprevalence, % (95% CI)
Overall	12,405	2,673	21.5 (20.8–22.3)	18.7 (15.1–22.9)	NA	18.4 (14.8–22.6)
Sex						
M	5,785	1,115	19.3 (18.3–20.3)	16.6 (13.2–20.6)	<0.001	16.3 (12.9–20.3)
F	6,493	1,538	23.7 (22.7–24.7)	20.6 (16.7–25.3)	Referent	20.3 (16.4–25.0)
Transgender	41	5	12.2 (4.1–26.2)	2.8 (0.2–27.6)	0.093	2.4 (0.0–27.3)
Age, y						
10–19	1,473	351	23.8 (21.7–26.1)	18.9 (14.7–24.0)	Referent	18.6 (14.4–23.7)
20–29	2,105	478	22.7 (20.9–24.6)	21.1 (16.8–26.2)	0.211	20.8 (16.5–25.9)
30–39	2,353	535	22.7 (21.1–24.5)	18.5 (14.6–23.1)	0.802	18.2 (14.3–22.8)
40–49	2,353	551	23.4 (21.7–25.2)	19.6 (15.5–24.5)	0.671	19.3 (15.2–24.2)
50–59	1,927	408	21.2 (19.4–23.1)	20.4 (16.1–25.5)	0.419	20.1 (15.8–25.2)
>60	2,108	335	15.9 (14.4–17.5)	13.4 (10.3–17.4)	0.001	13.1 (9.9–17.1)
History of respiratory symptoms						
Yes	175	114	65.1 (57.6–72.7)	59.8 (47.5–71.0)	<0.001	59.6 (47.3–70.9)
No	12,073	2529	20.9 (20.2–21.7)	18.3 (14.7–22.5)	Referent	18.0 (14.4–22.2)
Contact with COVID-19 case						
Yes	173	94	54.3 (46.6–61.9)	45.3 (34.6–56.6)	<0.001	45.1 (34.3–56.4)
No	11,938	2,498	20.9 (20.2–21.7)	18.3 (14.8–22.5)	Referent	18.0 (14.5–22.2)
Don’t know	137	51	37.2 (29.1–45.9)	22.1 (14.0–33.1)	0.363	21.8 (13.7–32.8)
Ever tested for COVID-19						
Yes	496	198	39.9 (35.6–44.3)	34.2 (26.9–42.5)	<0.001	33.9 (26.6–42.3)
No	11,752	2,445	20.8 (20.0–21.6)	18.0 (14.6–22.1)	Referent	17.7 (14.3–21.8)
COVID- 19 test result, n = 496						
Positive	119	107	89.9 (83.0–94.7)	NA	NA	NA
Negative	342	83	24.3 (19.8–29.2)	NA	NA	NA
Don’t Know	35	8	22.9 (10.4–40.1)	NA	NA	NA
*COVID-19, coronavirus disease; NA, not applicable; SARS-CoV-2, severe acute respiratory syndrome coronavirus 2.						

From our data, we estimated a total of 1,509,701 (95% CI 1,212,711–1,856,190) SARS-CoV-2 infections in Chennai. ICR per laboratory-confirmed case was 21.4 (95% CI 17.2–26.3) until July 7 and 19.2 (95% CI 15.4–23.6) until July 14, 2020.

## Conclusions

Our community-based survey indicated that ≈1/5 persons in Chennai was exposed to SARS-CoV-2 by July 2020. We noted a wide variation in the extent of infection across wards and seroprevalence ranged from 2%–50% (Appendix Table 3).

Seroprevalence was higher in northern Chennai and adjoining wards of central Chennai than in southern Chennai (Figure). Chennai witnessed a surge in COVID-19 cases in last week of April 2020 and >65% of cases were in northern Chennai (*7*). The number of cases showed a declining trend after the first week of July. Northern Chennai has a higher population density (55,000/km^2^) than Chennai (27,000/km^2^) and has several slum areas (*7*). High population density and persons living in close proximity might have contributed to the higher seroprevalence observed in northern Chennai.

**Figure:**
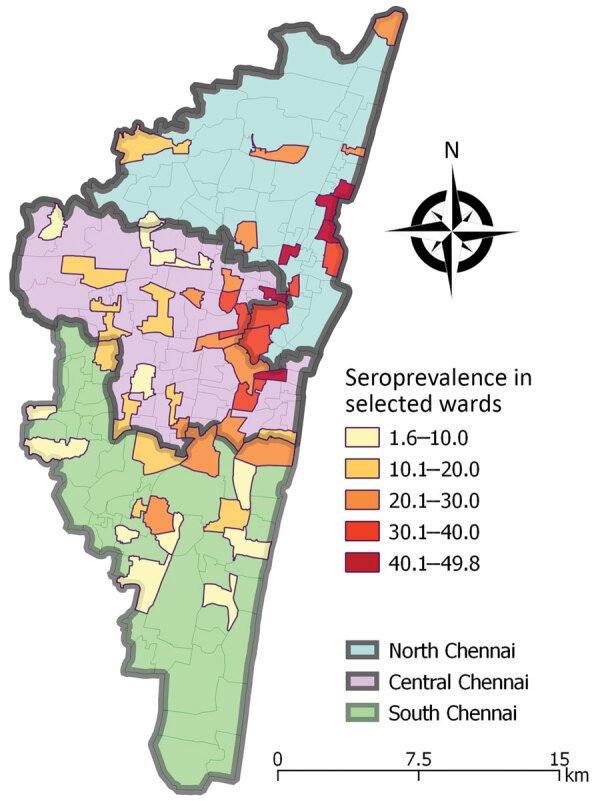
Seroprevalence of SARS-CoV-2 among residents of Chennai, India, July 2020. Values represent percent seroprevalence. SARS-CoV-2, severe acute respiratory syndrome coronavirus 2.

Seroprevalence was lower among male participants. Laboratory surveillance data in India showed a higher proportion of laboratory-confirmed COVID-19 among male than female patients (*8*). Comparable seroprevalence between children and adults suggests exposure within and outside of the household settings. Lower prevalence among persons >60 years of age could be due to lower exposure to infected persons or stricter adherence to nonpharmaceutical interventions. Serosurveys conducted in Santa Clara County, California, USA reported lower seropositivity among persons >60 years of age (E. Bendavid, et al. unpub. data, https://doi.org/10.1101/2020.04.14.20062463); however, in Spain, seropositivity was similar across all age groups (*9*) and in Greece, seroprevalence was higher among persons >60 years of age (*10*).

Most seropositive participants in our survey did not report any symptoms nor had any known contact with COVID-19 patient. IgG developed among most (107/119; 90%) recovered COVID-19 patients in our survey. Among 105 participants for whom >15 days had passed between RT-PCR confirmation of COVID-19 and blood sample collection for our serosurvey, 99 (94.2%) had seroconverted. Even after accounting for a 2-week delay for development of antibodies (*11*), ≈6% of COVID-19 patients were seronegative. Discordance between RT-PCR test results and presence of IgG might be due to poor B cell response or antibodies waning over time (*12*).

The ICR ranged from 19–21 and was lower than the ICR of 82–130 reported during the nationwide seroprevalence survey in India conducted in May 2020 (*5*). Lower ICR reflects a high level of case detection, resulting from extensive COVID-19 testing in the city. By July 15, 2020, Chennai had conducted 14,270 tests/million population.

Our study had 2 limitations. First, ≈1/3 persons from the visited households did not participate in the survey. Among them, 17.7% were not available at the time of visit and 13.5% refused to participate. Due to time constraints, we did not revisit households where persons were not available. The proportion of female participants and children 10–19 years of age was higher among persons who did not participate in the survey (Appendix Table 2), which might have influenced the seroprevalence estimates in either direction. Second, we might have underestimated the seroprevalence because antibodies to nucleocapsid protein have been shown to decline after infection (*13*).

In conclusion, ≈80% of the population in Chennai is still susceptible to SARS-CoV-2 infection. Transmission is expected to continue in wards with lower seroprevalence. Maintaining high testing rates and monitoring adherence to nonpharmacological interventions in GCC should be continued. In addition, periodic serosurveys would help monitor the trend of infection and assess the effects of varying containment measures in the city.

This study was funded by Greater Chennai Corporation public health department (PHDC no. 2797/20 dated July 9, 2020).

Appendix Additional information on a population-based serosurvey for SARS-CoV-2 among residents of Chennai, India, during July 2020.

## References

[1] World Health Organization. Coronavirus disease (COVID-19) situation report – 207 [cited 2020 Aug 15]. https://www.who.int/docs/default-source/coronaviruse/situation-reports/20200814-covid-19-sitrep-207.pdf

[2] State Control Room, Directorate of Public Health and Preventive Medicine Health and Family Welfare Department, Government of Tamil Nadu. Media bulletin 15.18.2020: daily report on public health measures taken for COVID-19 [cited 2020 Aug 15]. https://stopcorona.tn.gov.in/wp-content/uploads/2020/03/Media-Bulletin-15-08-20-COVID-19-6-PM.pdf.

[3] Greater Chennai Corporation. About Greater Chennai Corporation. [cited 2020 Sept 7]. https://www.chennaicorporation.gov.in/about-chennai-corporation/aboutCOC.htm

[4] Koopmans M, Haagmans B. Assessing the extent of SARS-CoV-2 circulation through serological studies. Nat Med. 2020;26:1171–2. 10.1038/s41591-020-1018-x 32719488

[5] Murhekar MV, Bhatnagar T, Selvaraju S, Rade K, Saravanakumar V, Vivian Thangaraj JW, et al. Prevalence of SARS-CoV-2 infection in India: Findings from the national serosurvey, May-June 2020. Indian J Med Res. 2020;152:48–60. 10.4103/ijmr.IJMR_3290_20 32952144 PMC7853249

[6] SARS-CoV-2 IgG immunoassay. Instructions for use. Abbott. May 2020 [cited 2020 Sep 07]. https://www.corelaboratory.abbott/us/en/offerings/segments/infectious-disease/sars-cov-2

[7] Special correspondent. Coronavirus: with over 65% of cases, all eyes on north Chennai. The Hindu. 2020 Apr 29 [citied 2020 Oct 26]. https://www.thehindu.com/news/cities/chennai/coronavirus-with-over-65-of-cases-all-eyes-on-north-chennai/article31467330.ece

[8] ICMR COVID Study Group. In alphabetical order: Abraham P, Aggarwal N, Babu GR, Barani S, Bhargava B, et al. Laboratory surveillance for SARS-CoV-2 in India: performance of testing & descriptive epidemiology of detected COVID-19, January 22–April 30, 2020. Indian J Med Res. 2020;151:424–437. 10.4103/ijmr.IJMR_1896_20 PMC7530445 32611914

[9] Pollán M, Pérez-Gómez B, Pastor-Barriuso R, Oteo J, Hernán MA, Pérez-Olmeda M, et al.; ENE-COVID Study Group. Prevalence of SARS-CoV-2 in Spain (ENE-COVID): a nationwide, population-based seroepidemiological study. Lancet. 2020;396:535–44. 10.1016/S0140-6736(20)31483-5 32645347 PMC7336131

[10] Bogogiannidou Z, Vontas A, Dadouli K, Kyritsi MA, Soteriades S, Nikoulis DJ, et al. Repeated leftover serosurvey of SARS-CoV-2 IgG antibodies, Greece, March and April 2020. Euro Surveill. 2020;25:2001369. 10.2807/1560-7917.ES.2020.25.31.2001369 32762796 PMC7459271

[11] Long QX, Liu BZ, Deng HJ, Wu GC, Deng K, Chen YK, et al. Antibody responses to SARS-CoV-2 in patients with COVID-19. Nat Med. 2020;26:845–8. 10.1038/s41591-020-0897-1 32350462

[12] Sekine T, Perez-Potti A, Rivera-Ballesteros O, Strålin K, Gorin JB, Olsson A, et al.; Karolinska COVID-19 Study Group. Robust T cell immunity in convalescent individuals with asymptomatic or mild COVID-19. Cell. 2020;183:158–168.e14. 10.1016/j.cell.2020.08.017 32979941 PMC7427556

[13] Ripperger TJ, Uhrlaub JL, Watanabe M, Wong R, Castaneda Y, Pizzato HA, et al. Orthogonal SARS-CoV-2 serological assays enable surveillance of low-prevalence communities and reveal durable humoral immunity. Immunity. 2020;53:925–933.e4. 10.1016/j.immuni.2020.10.004 33129373 PMC7554472

