# Supplementary material for: Population-Based Serosurvey for Severe Acute Respiratory Syndrome Coronavirus 2 Transmission, Chennai, India
Source: Emerg Infect Dis. 2021 Feb;27(2):586–9. doi: 10.3201/eid2702.203938 (PMC7853587; doi:10.3201/eid2702.203938)
Supplement: Appendix — Additional information on a population-based serosurvey for SARS-CoV-2 among residents of Chennai, India, during July 2020. [file 20-3938-Techapp-s1.pdf]

Article DOI: https://doi.org/10.3201/eid2702.203938

Population-Based Serosurvey for Severe Acute Respiratory Syndrome Coronavirus 2 Transmission, Chennai, India

Appendix

Laboratory Procedures

We tested 12,405 serum samples for the presence of IgG against severe acute respiratory syndrome coronavirus 2 (SARS-CoV-2) on the ARCHITECT i2000SR automated analyzer (Abbott, https://www.corelaboratory.abbott) by using the Abbott SARS-CoV-2 IgG immunoassay (*1*). The assay is a chemiluminescent microparticle immunoassay used for the qualitative detection of IgG to the nucleocapsid protein of SARS-CoV-2 in human serum and plasma. The sensitivity of the assay is 100% and specificity is 99.6% (*2*). The IgG in the sample binds to SARS-CoV-2 antigen-coated microparticles and undergoes a chemiluminescent reaction, producing a direct relationship between the amount of IgG and relative light units (RLU). The presence or absence of antibody in the sample is determined by comparing the RLU in the sample to the calibrator RLUs. The presence of antibody level above the cutoff index value $\geq$1.4 was interpreted as positive. Assay calibration was performed with positive and negative quality controls before analyses of samples. As a part of quality control, we retested 1% of the negative serum samples by using the same assay.

Statistical Analysis

The cross-sectional study considered 3 stages of sampling design. In the first stage, 51 wards were selected from among 200 wards in Chennai by using a probability proportional to size method. In the second stage, 6 streets were selected in each ward by using simple random sampling. The final stage was selecting the number of eligible persons in the household who agreed to participate in the survey.

We used a sampling fraction to compute the probability of selection at each stage of sampling. We computed design weights by the inverse of product of probabilities at all stages. The design weights were normalized and attached to the master dataset.

We used a random effects logistic regression model to address the clustering effect of estimates at all levels of hierarchical structure identified in the design. The hierarchical structure used in the analysis was ward, street, and household levels.

We modeled overall seroprevalence by using random intercept model for inclusion of each of the levels with design weights. We used the Akaike Information Criterion to select the final model. We also used this model to estimate seroprevalence for other factors, such as age and sex.

Seroprevalence estimates were obtained by exponentiating the log odds values obtained from the model and converting into probability to calculate corresponding 95% Wald confidence interval. We used the lme4 package from R software (R Foundation for Statistical Computing, https://www.r-project.org) to perform analysis.

We compared the weighted seroprevalence by selected demographic characteristics, history of respiratory symptoms, contact with laboratory-confirmed case of coronavirus disease, and coronavirus disease testing. We considered $p<0.05$ statistically significant.

We adjusted the weighted seroprevalence for test characteristics by using the following formula (*3*):

$$\text{Adjusted prevalence} = \frac{Weighted\ prevalence + specificity - 1}{Sensitivity + specificity - 1}$$

References

1. SARS-CoV-2 IgG immunoassay. Instructions for use. Abbott. May 2020 [cited 2020 Sep 07]. https://www.corelaboratory.abbott/us/en/offerings/segments/infectious-disease/sars-cov-2

2. Bryan A, Pepper G, Wener MH, Fink SL, Morishima C, Chaudhary A, Jerome KR, Mathias PC, Greninger AL. Performance characteristics of the Abbott Architect SARS-CoV-2 IgG assay and seroprevalence in Boise, Idaho. J Clin Microbiol. 2020;58:e00941–20. PubMed https://doi.org/10.1128/JCM.00941-20

3. Sempos CT, Tian L. Adjusting coronavirus prevalence estimates for laboratory test kit error. Am J of Epidemiol. 2020 Aug 17 [Epub ahead of print]. PubMed https://doi.org/10.1093/aje/kwaa174

Appendix Table 1. Selection of wards, streets, and households and number of persons in a serosurvey for SARS-CoV-2, Greater Chennai Corporation, India, July 2020*

		No. Wards		No. streets		No. households		No. persons		
Code	Zone	Total	Selected	Total	Selected	Surveyed	Responded (%)	Eligible	Available (%)†	Enrolled (%)‡
1	Thiruvottriyur	14	3	1,290	22	438	416 (95.0)	907	756 (83.4)	654 (86.5)
2	Manali	7	1	1,446	6	164	162 (98.8)	421	271 (64.4)	245 (90.4)
3	Madhavaram	13	2	1,831	12	295	271 (91.9)	737	597 (81.0)	368 (61.6)
4	Tondiarpet	14	4	3,071	29	682	650 (95.3)	1,605	1,181 (73.6)	866 (73.3)
5	Royapuram	15	4	1,596	24	598	493 (82.4)	1,315	1,130 (85.9)	945 (83.6)
6	Thiru-Vi-Ka Nagar	14	5	2,712	31	849	709 (83.5)	1,816	1,477 (81.3)	1,085 (73.5)
7	Ambattur	15	4	3,541	24	536	462 (86.2)	1,361	1,157 (85.0)	984 (85.0)
8	Anna Nagar	16	4	2,645	24	719	538 (74.8)	1,496	1,244 (83.2)	974 (78.3)
9	Teynampet	18	5	1,973	35	747	731 (97.9)	1,605	1,332 (83.0)	1,236 (92.8)
10	Kodambakkam	15	4	2,575	24	572	539 (94.2)	1,270	1,122 (88.3)	990 (88.2)
11	Valasarvakkam	13	2	6,675	12	353	344 (97.5)	724	578 (79.8)	522 (90.3)
12	Alandur	12	2	2,047	12	306	281 (91.8)	747	649 (86.9)	510 (78.6)
13	Adyar	13	7	5,701	42	1,058	1,026 (97.0)	2,537	2,056 (81.0)	1,855 (90.2)
14	Perunkudi	11	3	3,115	18	460	454 (98.7)	1,051	957 (91.1)	890 (93.0)
15	Sozhinganallur	10	1	2,392	6	158	158 (100.0)	448	332 (74.1)	281 (84.6)
Total		200	51	42,610	321	7,935	7,234 (91.2)	18,040	14,839 (82.3)	12,405 (83.6)

*SARS-CoV-2, severe acute respiratory syndrome coronavirus 2.
†Percentage calculated out of eligible participants.
‡Percentage calculated out of available participants.

Appendix Table 2. Seroprevalence of IgG antibodies against SARS-CoV-2 by selected wards, Greater Chennai Corporation, India, July 2020*

	No. persons		Unweighted seroprevalence,	Test performance adjusted
Ward Name	Tested	Positive	% (95% CI)	seroprevalence, % (95% CI)
Ward 47–Korukkupet	245	123	50.0 (43.7–56.3)	49.8 (43.5–56.1)
Ward 39–New Washermen Pet	230	113	49.0 (42.5–55.5)	48.8 (42.3–55.3)
Ward 14–Kaladipet	200	94	47.0 (40.1–53.9)	46.8 (39.8–53.7)
Ward 43–Royapuram	243	115	47.0 (40.7–53.3)	46.8 (40.5–53.1)
Ward 115–Royapettah	240	106	44.0 (37.7–50.3)	43.8 (37.5–50.1)
Ward 77–Pulianthope	157	68	43.0 (35.3–50.7)	42.8 (35.0–50.5)
Ward 104–Egmore	242	97	40.0 (33.8–46.2)	39.8 (33.6–46.0)
Ward 61–Egmore	227	83	37.0 (30.7–43.3)	36.7 (30.4–43.1)
Ward 54–Kondithoppu	219	76	35.0 (28.7–41.3)	34.7 (28.4–41.1)
Ward 50–Royapuram	252	84	33.0 (26.2–39.8)	32.7 (25.9–39.5)
Ward 58–Choolai	247	82	33.0 (27.1–38.9)	32.7 (26.8–38.6)
Ward 74–Nammalwarpet	186	61	33.0 (27.1–38.9)	32.7 (26.8–38.6)
Ward 118–Teynampet	247	81	33.0 (27.2–38.8)	32.7 (26.9–38.6)
Ward 111–Royapettah	246	68	28.0 (22.4–33.6)	27.7 (22.1–33.3)
Ward 8–Tiruvottriyur	249	66	27.0 (21.4–32.6)	26.7 (21.1–32.3)
Ward 71–Otteri	241	64	27.0 (21.5–32.5)	26.7 (21.1–32.3)
Ward 171–Saidapet	246	66	27.0 (21.5–32.5)	26.7 (21.2–32.2)
Ward 122–Raja Annamalai Puram	249	64	26.0 (20.6–31.4)	25.7 (20.2–31.2)
Ward 173–Raja Annamalai Puram	259	64	25.0 (19.7–30.3)	24.7 (19.4–30.0)
Ward 21–Manali	245	59	24.0 (18.7–29.3)	23.7 (18.3–29.1)
Ward 1–Kathivakkam	205	47	23.0 (17.2–28.8)	22.7 (16.9–28.5)
Ward 97–Ayanavaram	241	56	23.0 (17.7–28.3)	22.7 (17.4–28.0)
Ward 140–Saidapet	261	60	23.0 (17.9–28.1)	22.7 (17.6–27.8)
Ward 177–Velachery West	274	62	23.0 (18.0–28.0)	22.7 (17.7–27.7)
Ward 36–Vyasarpadi	148	32	22.0 (15.3–28.7)	21.7 (15.0–28.4)
Ward 107–Chetpet	251	56	22.0 (16.9–27.1)	21.7 (16.5–26.8)
Ward 163–Adambakkam	240	49	20.0 (14.9–25.1)	19.7 (14.6–24.8)
Ward 93–Mugappair East	247	42	17.0 (12.3–21.7)	16.7 (12.0–21.4)
Ward 126–Raja Annamalai Puram	254	44	17.0 (12.4–21.6)	16.7 (12.0–21.3)
Ward 145–Nerkundram	269	47	17.0 (12.5–21.5)	16.7 (12.1–21.2)
Ward 170–Ekkattuthangal	266	45	17.0 (12.5–21.5)	16.7 (12.2–21.2)
Ward 84–Korattur	237	38	16.0 (11.3–20.7)	15.7 (11.0–20.3)
Ward 180–Thiruvanmiyur	273	44	16.0 (11.7–20.3)	15.7 (11.3–20.0)
Ward 100–Anna Nagar	240	36	15.0 (10.5–19.5)	14.7 (10.1–19.2)
Ward 133–West Mambalam	244	37	15.0 (10.5–19.5)	14.7 (10.2–19.2)
Ward 89–A.N.W. Extension	254	36	14.0 (9.7–18.3)	13.7 (9.4–17.9)
Ward 22–Kavankarai	203	22	11.0 (6.7–15.3)	10.6 (6.3–15.0)
Ward 137–Nesapakkam	236	26	11.0 (7.0–15.0)	10.6 (6.6–14.7)
Ward 67–Peravallur	246	24	10.0 (6.3–13.7)	9.6 (5.9–13.4)
Ward 129–Saligramam	249	25	10.0 (6.3–13.7)	9.6 (5.9–13.4)
Ward 150–Karambakkam	253	26	10.0 (6.3–13.7)	9.6 (5.9–13.4)
Ward 193–Thoraipakkam	281	29	10.0 (6.5–13.5)	9.6 (6.1–13.2)
Ward 65–Kolathur	255	23	9.0 (5.5–12.5)	8.6 (5.1–12.2)
Ward 178–Velachery East	260	24	9.0 (5.5–12.5)	8.6 (5.1–12.1)

Ward Name	No. persons Tested	No. persons Positive	Unweighted seroprevalence, % (95% CI)	Test performance adjusted seroprevalence, % (95% CI)
Ward 183–Kottivakkam	261	23	9.0 (5.5–12.5)	8.6 (5.1–12.1)
Ward 80–Pudur	246	17	7.0 (3.8–10.2)	6.6 (3.4–9.8)
Ward 188–Madipakkam	350	24	7.0 (4.3–9.7)	6.6 (3.9–9.3)
Ward 168–Ullagaram	279	18	6.0 (3.2–8.8)	5.6 (2.8–8.4)
Ward 175–Thiruvanmiyur	277	15	5.0 (2.4–7.6)	4.6 (2.0–7.2)
Ward 156–Mugalivakkam	270	8	3.0 (1.0–5.0)	2.6 (0.6–4.7)
Ward 32–Lakshmipuram–Madhavaram	165	4	2.0 (0.0–4.1)	1.6 (0.0–3.8)

*SARS-CoV-2, severe acute respiratory syndrome coronavirus 2.

Appendix Table 3. Characteristics of persons surveyed for enrollment in severe acute respiratory syndrome coronavirus 2 serosurvey, Greater Chennai Corporation, India, July 2020

Characteristics	Participated in the survey, no. (% of total)	Refused to participate in serosurvey, no. (% of total)
Total	12,319	2,434
Age, y*		
10–19	1,473 (12.0)	522 (21.4)
20–29	2,105 (17.1)	468 (19.2)
30–39	2,353 (19.1)	406 (16.7)
40–49	2,353 (19.1)	364 (15.0)
50–59	1,927 (15.6)	302 (12.4)
$\geq$60	2,108 (17.1)	372 (15.3)
Sex**		
M	5,785 (47.0)	1,015 (41.7)
F	6,493 (52.7)	1,412 (58.0)
Other	41 (0.3)	7 (0.3)

*χ^2 test comparing those participated and those who refused, 184.12 (p<0.001).
**χ^2 test comparing those participated and those who refused, 22.9 (p<0.001).
